# 

**Published:** 1871-01

**Authors:** 


### INDEX TO VOLUME XXV.

Address ---------- 18
A New Preparation of Cotton for Stanching Hemorrhage ---------- 37
Antimonoid ---------- 38
A Legion of Leeches ---------- 42
An Itemized Bill ---------- 47
Ability vs. Compensation ---------- 108
Are the Mineral Acids Formed in the Mouth? ---------- 111
Associative Effort ---------- 114
A Peculiar Case ---------- 117
A Cure for Love Melancholy ---------- 118
A Reversion Spectroscope ---------- 139
A War Victim ---------- 139
Address to the Graduating Class ----------143, 243
Advantage of Clinics ---------- 160
A Lapse ---------- 186
Alleged Cause of Death from Nitrous Oxyd ---------- 224
Are the Mineral Acids Formed in the Mouth? ---------- 413
American Dental Association ---------- 411
Address before the Alumni Association ---------- 264
A New Impression Cup ---------- 272
A Pumice Polisher ---------- 273

Address read before the Northern Ohio Dental Association---------- 301
A Case in Practice---------------------------------------------- 304
A New Experience ---------------------------------------------- 350
Atrophy of the Teeth-------------------------------------------- 352
American Dental Association------------------------------------- 411
A Case, and its Perplexities ------------------------------------ 461
Annual Meeting of the Lake Erie Dental Association ------------- 466
Anæsthesia ---------------------------------------------------- 511
Are We Progressing?--------------------------------------------- 514
Bee Stings----------------------------------------------------- 38
Bone Phosphate------------------------------------------------- 49
Boston Society of Medical Sciences------------------------------ 86
California State Dental Association----------------------------- 361
Crude Teachings ----------------------------------------------- 406
Change and Removal--------------------------------------------- 453
Correspondence ------------------------------------------------ 453
Corns on the Feet---------------------------------------------- 48
Chemical Nature of Malaria------------------------------------- 501
Change Editorial----------------------------------------------- 543
Close of the Volume-------------------------------------------- 543
Dental Hygiene------------------------------------------------- 57
Deaths from Chloroform ---------------------------------------- 91
Discussions of the 27th Meeting of Miss. Valley Dental Society---- 163
Dental Education---------------------------------------199, 235, 454
Death of a Noted Southern Physician----------------------------- 234
Demonstrations vs. Lectures------------------------------------- 253
Dental Physiology---------------------------------------------- 281
Dental Organization in Kansas ---------------------------------- 321
Dental Jottings-------------------------------------------333, 457
Dental Ethics------------------------------------------341, 391, 427
Dentistry vs. Money-------------------------------------------- 379
Dentigerous Cysts---------------------------------------------- 532

Disease of the Antrum ........ 540
Eau de Beaule de M. Bargasse ........ 92
Eighth District Society of New York ........ 275
Educational ........ 276
Examination ........ 407
Extracts from Proceedings of the Forest City Society of Dental Surgeons ........ 40[illegible]
Eastern Ohio Dental Association ........ 497
East Tennessee Dental Association ........ 529
Eastern Indiana Dental Society ........ 531
Fifth Annual Meeting of the Ohio Dental Society ........ 64
First Dentition ........ 189
Filling Teeth ........ 236
First District Dental Society of the State of New York ........ 521
Gas vs. Chloroform ........ 106
How Not to Do It ........ 94
Hygienic Treatment of Disease ........ 90
How to Spell and Pronounce Aluminium ........ 514
Irregular Dentition ........ 39
Immobility of the Jaws ........ 45
Investigations of the Tooth Pulp ........ 97
Influence of Diet on the Composition of Bone ........ 118
In Memoriam—Dr. J. G. Willis ........ 280
Insanity in Woman ........ 323
Indications for Extraction of the Teeth ........ 325
Is it a Third Tooth, or the Development of the Long Dormant Pulp of a Second Molar? ........ 463
Kentucky State Dental Society ........ 544
Loss of Speech After Chloroform ........ 38
Lancing the Gums ........ 134
Little Things ........ 182
Meetings ........ 51

Minutes of the Fifth Annual Meeting of the Ohio State Dental Society, Columbus, Ohio 122
Medical Education 130
Mistaken Impressions on Nitrous Oxyd 110
Mechanical Dentistry 154
Mississippi Valley Dental Society 183
May Fillings be Made Too Dense? 247
Minutes of the Wabash Valley Dental Association 260
Minutes of the 13th Annual Indiana State Dental Association 394
Mad River Dental Society 452
Material Supplies for Our Profession 500
Neuralgia of the Jaw Bones 41
Neuralgic Pill 47
Nervous System 53
New Jersey State Dental Society 119
Note on the Physiological Effects of Carbonic Oxide 180
Nitrous Oxyd Rumors 232
Northern Ohio Dental Association 311
Necrosis of the Lower Jaws 335
Ohio State Dental Society—Discussions 22
Oxygen Gas Prepared at the Ordinary Temperature 47
Office Notes 61, 104, 252
Offensive Breath 132
Obituaries 188
Oxychloride of Zinc Treatment of Dental Pulps 256
Oblivious 360
Oxygen in its Relationship to the Vital Phenomena 369
Obituary 412
Operative Dentistry—or, Filling Teeth 437
Physiology of the Pancreatic Secretion 36
Parchment Paper 48
Preservation of Anatomical Specimens 88

Proceedings of Societies. 64, 119, 163, 226, 260, 311, 361, 394, 444, 466, 516
Professional Responsibility 93
Pneumatic Engine 95
Puffery 105
Phosphate of Lime 186, 250
Proceedings of the 27th Annual Meeting of the Mississippi Valley Dental Society 226
Preservation of the Cadaver 229
Professional Fidelity 290
Phrenology and Dentistry 295
Professor Tyndall on the Chemistry of Light—New Experiments and Conclusions 306
Proceedings of the Wisconsin State Dental Society 444
Proceedings of the Kansas State Dental Society 484
Proceedings of the Michigan Central Dental Association 491
Proceedings of the Brooklyn Dental Society 528
Proceedings of the Tennessee Dental Society 530
Selections 34, 86, 118, 179, 264
Selections from a Clinical Lecture on Tumors 34
Simple Method of Arresting Obstinate Epistaxis 38
Skin Grafting 44
Speaking and Singing Without a Tongue 91
Splitting Teeth with the Mallet 116
Styptic Wool 137
Surgery that is Surgery 320
Southern Dental Association 497
State Society 499
Second Annual Meeting of the New Jersey State Dental Society 516
Thoughts on Respiration 9
Tic Doloreux Cured by Galvanism 40
Table of Societies 51
The Forthcoming Volume 50

The Philosophy of Chills and Fever ........ 138
The Discrimination between Syphilis and Scrofula ........ 138
Two Very Perplexing Cases ........ 158
Things Old and New ........ 161
The Microscope in Medicine ........ 179
The Production of Asthenia or Anæsthesia in Surgical Operations by Compression of the Vagus Nerves ........ 181
The Treatment of Suppurating Glands of the Neck ........ 187
To the Point ........ 234
The Mad River Dental Society ........ 272
The Northern Ohio Dental Association ........ 273
The Hot Air Syringe ........ 274
The Law ........ 279
The Physiology of Thought ........ 319
The Pneumatic Engine Improvement ........ 8[illegible]
The Brooklyn Dental Society ........ 4[illegible]
The Chicago Fire ........ [illegible]
The State Board of Dental Examiners ........ 4[illegible]
The Ohio State Dental Society ........ 54[illegible]
Watt's Elevator—A Misnomer ........ 18[illegible]
When Philosophers Differ ........ 368
Vital Phenomena ........ 214

### THE FORTHCOMING VOLUME

Will present some improvements, we trust, which will be acceptable to our subscribers. A better quality of paper is used—it is a fine book paper—the typographical execution will present improvement, and in other respects it is expected the profession will aid in securing constant improvement. Send in well written communications. T.

### MEETINGS.

The Mississippi Valley Dental Society will hold its annual meeting, beginning on Wednesday, at 10 o'clock, A. M., the 1st of March next. It is unnecessary for us to say anything to the old members in the way of urging them to come, for they know that this is one of the most efficient dental societies that has ever been organized; and they know when they come up to its meetings what to expect, and we are confident they will all so far as possible be present. And to all who have not met with this Society we same COME, and it will do you good,—you will lose nothing, but gain much. Let every one come prepared to present something; either a few thoughts, *or a thought*, or some valuable mode of operation, instrument or appliance. A very certain way of receiving something valuable is to bring something of value.

Two questions usually and should always occur to us after attending our professional meetings, viz.: Have I received anything valuable? and, have I given anything of real worth to others? With the first of these most persons seem to be most concerned; they regard every meeting from which they have not gained some especial new and valuable idea, as a failure—rather a sad reflection, to say the least, upon their ability to do good to others, which is quite as important a matter as to receive.

Let all come prepared to contribute something, and let this, the twenty-seventh annual meeting of the Mississippi Valley Dental Society be one of the best ever held. T.

---

The Ohio Dental College Association will meet on Tuesday, Feb. 28th, at 2 o'clock, P. M., when it is very desirable that all the members should be present, as very important business will come before the Association for consideration and decision, in settling which every member should have a voice. Then let every member so far as possible be in attendance. T.

---

At the meeting of the State Board of Dental Examiners held in Columbus, in December, the following members of the profession presented themselves, and having passed a satisfactory examination, received each a certificate of qualification to practice dentistry in the State of Ohio, viz: E. Chidester, Massillon; J. H. Siddal, Canton; T. J. Fahnestock, Cincinnati; J. S. McCarrall, Bucyrus; W. J. Moffit, Coshocton; M. A. Spencer, Orrville; G. A. Curtis, Galion; M. Jefferson, Cambridge; S. B. Lewis, Greenville; W. T. Semple, Mt. Vernon: Robt. W. Stevens, Mt. Vernon. Most of these were men of many years' experience in the profession, and had already made a good reputation, but having a commendable regard for the law, and the best interests of the profession, sought an early opportunity to comply with the requirements. The next meeting of the Board will be held in Cleveland, on Wednesday the 7th of June next.

---

### NEW FOIL.

We have been using a foil just introduced, bearing the name of Geo. Watt & Co. as manufacturers, better than which we have never used. It is soft and pliable, yet very adhesive. It seems to have about every quality that can be expected in gold foil. We advise all to try it. So far as we have tested it it is very uniform in working quality. T.

### OHIO COLLEGE

OF

### DENTAL SURGERY.

The Twenty-sixth Annual Session of the Ohio College of Dental Surgery will commence on Tuesday the 17th of October next, and continue until March 1st, '72.

### FACULTY.

JAMES TAYLOR, M. D., D. D. S.,
Emeritus Professor of Institutes of Dental Science.

C. KEARNS, M. D.,
Professor of Anatomy.

D. VAUGHAN,
Professor of Chemistry and Therapeutics.

EDWARD RIVES, M. D.,
Professor of Physiology and Histology.

F. BRUNNING, M. D.,
Professor of Pathology and Therapeutics.

J. TAFT, D. D. S.,
Professor of Operative Dentistry and Dental Hygiene.

C. M. WRIGHT, D. D. S.,
Professor of Mechanical Dentistry and Metallurgy.

WILL TAFT, D. D. S.,
Professor of Clinical Dentistry.

A. SCHWAGMEYER, M. D.
Demonstrator of Anatomy.

### TEXT BOOKS.

Gray's and Wilson's Anatomy; Dalton's or Carpenter's Physiology; Williams' Pathology; Fown's, Turner's, Graham's, or Brandt and Taylor's Chemistry; Taft's Operative Dentistry; Richardson's Mechanical Dentistry; Harris' Principles and Practice of Dental Science, Tomes' Dental Surgery, Watts' Chemical Essays, Harris' Dental Dictionary.

### TERMS OF ADMISSION.

Tickets must be procured at the beginning of the session.

| | |
|---|---|
| Tickets for the course, | $100 00 |
| Demonstrator's Ticket for Anatomy, | 5 00 |
| Matriculation Fee, but once, | 5 00 |
| Diploma Fee, | 30 00 |

### TERMS OF GRADUATION.

Two courses, (including one of dissections,) with a satisfactory dental pupilage.

Eight years reputable practice, or a satisfactory examination upon the branches of the Junior Course in this Institution will be received as equal to one course.

An acceptable thesis upon some subject on Dental science.

Must possess a good moral character, and be twenty-one years old.

For further information, address J. TAFT, *Dean.*
117 West Fourth Street.

### CONTENTS.

ORIGINAL COMMUNICATIONS.

Thoughts on Respiration .......... 9

Address .......... 18

Ohio State Dental Society—Discussions .......... 22

SELECTIONS.

Selections from a Clinical Lecture on Tumors .......... 34

Physiology of the Pancreatic Secretion .......... 36

A New Preparation of Cotton for Stanching Hemorrhage .......... 37

Loss of Speech After Chloroform .......... 38

Simple Method of Arresting Obstinate Epistaxis .......... 38

Bee Stings .......... 38

Antimonoid .......... 38

Irregular Dentition .......... 39

Tic Douloureux Cured by Galvanism .......... 40

Neuralgia of the Jaw Bones .......... 41

A Legion of Leeches .......... 42

Skin Grafting .......... 44

Immobility of the Jaws .......... 45

An Itemized Bill .......... 47

Neuralgic Pill .......... 47

Oxygen Gas Prepared at the Ordinary Temperature .......... 47

Corns on the Feet .......... 48

Parchment Paper .......... 48

EDITORIAL.

Bone Phosphate .......... 49

The Forthcoming Volume .......... 50

Table of Societies .......... 51

Meetings .......... 51

New Foil .......... 52

---

### The Dental Register

### OF THE WEST,

*Issued Monthly, at $3 a Year in Advance.*

DEVOTED TO THE INTERESTS OF THE DENTAL PROFESSION.

EDITED BY J. TAFT AND G. WATT.

The volume commences in January and closes in December.

The Register being issued monthly is always fresh, therefore indispensable to the practitioner who desires to keep posted up with the new inventions and improvements which are daily developed. Neither expense nor effort will be spared to give value and variety to its contents. Articles will be illustrated with well executed engravings whenever they will add to the interest or assist in explanation.

Its extensive circulation and frequency of issue makes it one of the BEST DENTAL ADVERTISING MEDIUMS in the United States.

RATES OF ADVERTISING.

One page, 1 year, $50. Half page, 1 year, $30. Quarter page, or card, 1 year $20.

All advertising bills payable in advance, or at furthest after the issue of the first number containing the advertisement. All transient advertisements to be paid for in advance.

☞ CONTRIBUTIONS SOLICITED.

Address, J. TAFT, or "DENTAL REGISTER," Cincinnati Ohio.

Business Communications to

**J. TAFT, Publisher,**

No. 117 West Fourth St.

